# Factors associated with financial toxicity in patients with breast cancer in Japan: a comparison of patient and physician perspectives

**DOI:** 10.1007/s12282-023-01476-z

**Published:** 2023-06-13

**Authors:** Sumito Saeki, Tsuguo Iwatani, Atsuko Kitano, Naomi Sakurai, Yuko Tanabe, Chikako Yamauchi, Ataru Igarashi, Yusuke Kajimoto, Sayaka Kuba, Fumikata Hara, Yasuaki Sagara, Shinji Ohno, Collaborative Study Group of Scientific Research of the Japanese Breast Cancer Society

**Affiliations:** 1 Breast Oncology Center, Cancer Institute Hospital, Japanese Foundation for Cancer Research, 3-8-31 Ariake, Koto-ku, Tokyo, 135-8550 Japan; 2 Graduate School of Medicine, Department of Cancer Therapy and Surgery, Tohoku University, Sendai, Japan; 3 Department of Breast Surgery, National Cancer Center Hospital East, Chiba, Japan; 4 Department of Medical Oncology, St Luke’s International Hospital, Tokyo, Japan; 5 Cancer Solutions, Inc, Tokyo, Japan; 6 Department of Medical Oncology, Toranomon Hospital, Tokyo, Japan; 7 Radiation Therapy Center, Shiga General Hospital, Moriyama, Japan; 8 Department of Pharmaceutical Policy, The University of Tokyo, Tokyo, Japan; 9 Graduate School of Pharmaceutical Sciences, University of Tokyo, Tokyo, Japan; 10 Oncology Science Unit, MSD K.K, Tokyo, Japan; 11 Graduate School of Biomedical Sciences, Department of Surgery, Nagasaki University, Nagasaki, Japan; 12 Department of Breast and Thyroid Surgical Oncology, Hakuaikai Sagara Hospital, Kagoshima, Japan

**Keywords:** Financial burden, Breast neoplasms, Health services accessibility, Multivariate analysis, Collaborative study group of scientific research of the Japanese Breast Cancer Society

## Abstract

**Background:**

Financial toxicity (FT) is a notable concern for patients with breast cancer worldwide. The situation regarding FT in Japan, however, has not been well explored. This study examined FT in patients with breast cancer in Japan and presented an overview of the group study’s overall findings.

**Methods:**

The survey used the Questant application and primarily targeted patients with breast cancer attending research facilities and physicians who are members of the Japanese Breast Cancer Society. The Japanese version of the Comprehensive Score for FT (COST) was used to quantify patients’ FT. Multiple regression analysis was used to identify factors related to FT in patients with breast cancer in Japan and evaluate the sufficiency of information support level (ISL) for medical expenses.

**Results:**

We collected 1558 responses from patients and 825 from physicians. In terms of factors affecting FT, recent payments had the highest impact, followed by stage, and related departments positively affecting FT. Conversely, factors such as income, age, and family support were found to negatively affect FT. A significant discrepancy was identified between patients and physicians in perceived information support, with patients frequently feeling unsupported and physicians believing that they have provided adequate support. Furthermore, differences in the frequency of explanations and opportunities to ask questions about medical costs across FT grades were found. The analysis also showed that physicians with a better understanding of information support needs and greater knowledge of medical costs tended to provide more support that is comprehensive.

**Conclusion:**

This study emphasizes the importance of addressing FT in patients with breast cancer in Japan and highlights the need for enhanced information support, deeper understanding by physicians, and collaborative efforts among professionals to mitigate financial burden and provide personalized, tailored support for individual needs.

**Supplementary Information:**

The online version contains supplementary material available at 10.1007/s12282-023-01476-z.

## Introduction

The number of patients diagnosed with breast cancer is increasing annually [1, 2], emphasizing the need for improved screening accuracy [3, 4], comprehensive treatment methods, and personalized treatments [2, 5]. However, patients with breast cancer tend to be younger than those with other cancers [6, 7], resulting in direct burdens, such as longer treatment periods and increased costs [8, 9], as well as indirect burdens, such as adverse event treatments [10], fertility preservation [11, 12], and lost earnings [13]. Financial toxicity (FT) is a notable issue for patients with breast cancer globally [14–16], with the lack of medical cost information support for patients with breast cancer in the United States being a notable concern [17–20]. In Japan, there have been reports about the relationship between FT and Quality of Life in gynecological cancer patients [21], but the actual situation and issues of information support regarding FT and medical costs for patients with breast cancer are unclear. Therefore, this study aims to identify factors related to FT in patients with breast cancer in Japan, evaluate the sufficiency of information support related to medical expenses, and provide an overview of the group study’s overall findings in parallel with other related studies.

## Methods

### Patient survey

**Fig. 1:**
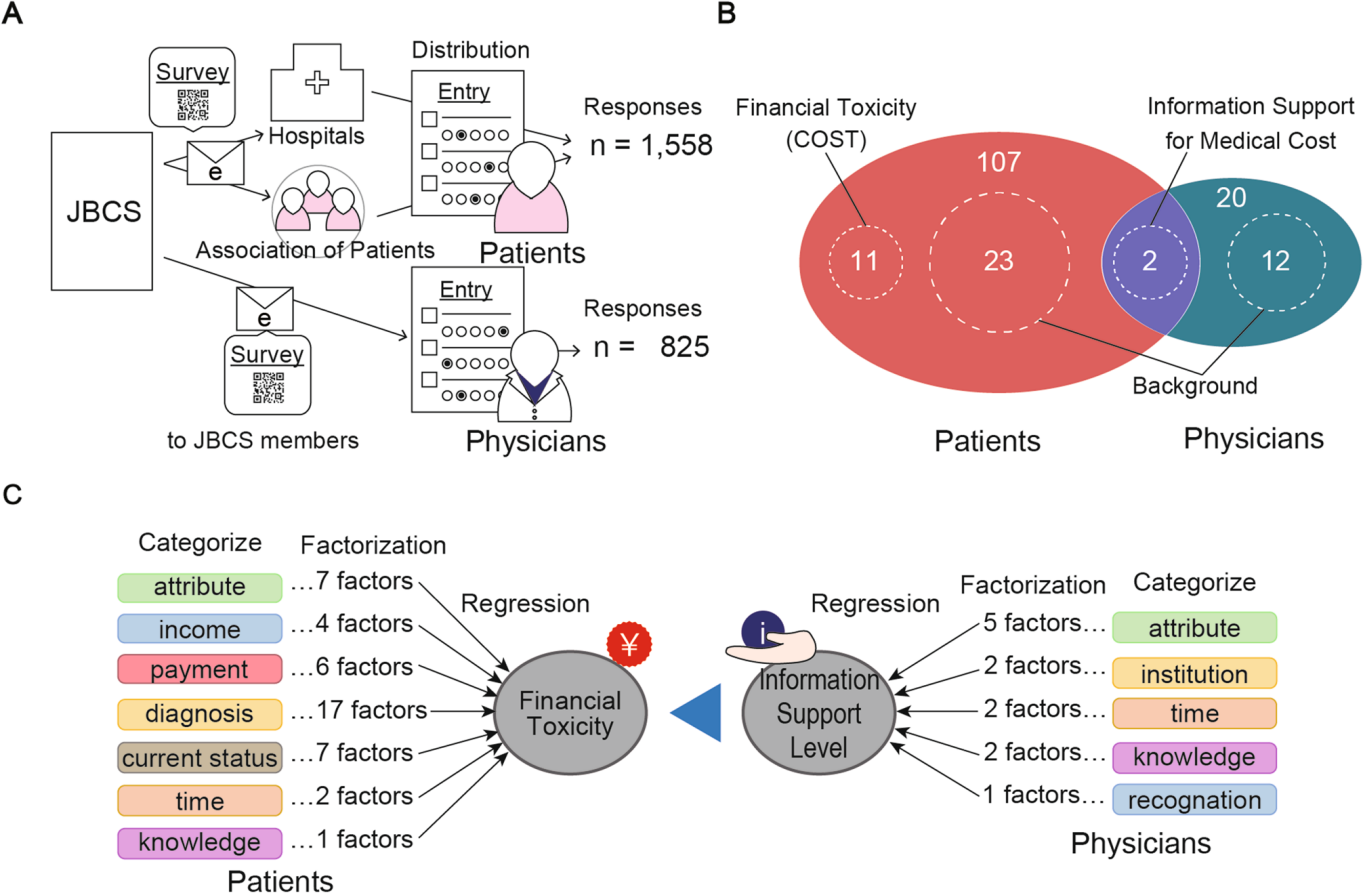
Study overview. **A** The questionnaire method and the number of responses from patients and physicians. **B** The number of questionnaire items used in the study for each group. **C** Classification of patient factors underlying Financial toxicity and physician factors behind information support

This study included patients who had been treated for breast cancer, were either inpatients or outpatients at collaborating institutions or had completed their treatment (remission). Consent was obtained before they participated in the study. The survey was conducted across 18 institutions in Japan, consisting of 10 primary hospitals and 8 clinics, encompassing a diverse range of academic hospitals and regional core facilities (exact names are not disclosed in the manuscript). The surveyed institutions were geographically widespread across the nation, except for the Chubu region where there were no facilities with enrolled group members. Based on the projected response rate, we aimed to engage 2000 patients from the participating facilities. The survey was conducted from December 2020 to April 2021. Patients were provided the QR code required to access the application in person, through posters in hospitals, and distribution to patient groups and social networking sites, thus extending our reach beyond traditional healthcare facilities (Fig. 1A). In collaboration with the Japanese Breast Cancer Society (JBCS) group study members, a comprehensive questionnaire was developed, consisting of 107 questions for patients (Fig. 1B). The survey was administered using the Questant application (MACROMILL, INC., Tokyo, Japan). The questionnaire included 23 questions specifically related to the patient’s background. These questions were categorized into seven groups, followed by principal component analysis (PCA) (Fig. 1C) using the methods described below. All procedures involving human participants complied with the ethical standards of the institutional research committees and with the 1964 Declaration of Helsinki and its later amendments.

### Physician survey

The study involved physicians who were members of the JBCS, including various specialties. They were invited to participate through an official email from the JBCS (Fig. 1A). The physician survey followed the same format as the patient survey. Based on the number of members in the JBCS, the target participant count was set at 500 physicians. Direct patient-physician correspondence was not a requirement for this study. The questionnaire for physicians consisted of 20 questions, including 12 questions specifically related to their backgrounds (Fig. 1B). These questions were categorized into five groups, and only those with high correlations were subjected to PCA (Fig. 1C). However, for geographic data, PCA was used to combine latitude and longitude into a singular measure, termed “location (longitude)”. For further examination, multiple comparisons were anticipated to ascertain if the level of information support varied by physician specialty, a measure which is elaborated on in the following sections.

### Survey on patients’ and physicians’ attitudes

To evaluate the information support for medical costs, two shared questions were directed to both patients and physicians. These inquiries focused on the extent of information provided and the opportunities for questions. Responses were collected on a five-point Likert scale (1 = not at all, 2 = somewhat agree, 3 = agree, 4 = fairly agree, 5 = strongly agree). We evaluated the differences in these indicators between patients, physicians, as well as among the various grades of FT. Given that these questions relate to the level of information support for medical costs, and they could be consolidated into a single measure, we extracted the first principal component (PC) from both questions and termed it the information support level (ISL). To explore regional differences in ISL, we computed prefecture-specific averages for both patients and physicians and represented these data as a heat map on a geographical layout. Further, we conducted multiple comparisons to identify potential differences in ISL across different prefectures.

### Scale conversion, parameter setting, and data classification

The ranking scales, comprising the choices, were converted to continuous values, and the nominal scales included in the choices were transformed into independent scales. The longitudes and latitudes of the locations of patients’ places of residence and physicians’ offices were extracted from the Geospatial Information Authority of Japan website [22]. Population data were extracted from the Statistics Bureau, Ministry of Internal Affairs and Communications website [23]. A PCA was performed for each group, which was categorized into several factors. PCs were limited to those with eigenvalue (EV) > 1 or a cumulative contribution ratio (CCR) > 80% and those that could be assigned meaning (Fig. 1C).

### Definition of financial toxicity (FT)

The Japanese version of the Comprehensive Score for Financial Toxicity (COST) was utilized to quantify patients’ FT [24, 25]. The COST scale, according to the developer’s definition, produces a continuous variable ranging from 0 to 44. Smaller values signify a higher degree of FT [26–28], resulting in the following formula for FT calculation: FT = 44 − COST. Additionally, FT was stratified into four grades based on the developer’s guidance: G0, no FT, COST ≥ 26; G1, mild FT, COST between 14 and 26; G2, moderate FT, COST > 0 and up to 14; and G3, severe FT, COST = 0 [25].

### Data analysis

All analyses were performed using JMP® 17.0.0 (SAS Institute Inc., Cary, NC, USA). To evaluate differences in patients’ and physician’ attitudes concerning the level of information support, as well as variations in patients’ attitudes based on FT grades, Wilcoxon rank-sum tests were employed. Kruskal–Wallis tests were utilized for multiple comparisons of ISL by prefectures for both patients and physicians and by specialty for physicians. When significant differences emerged, pairwise comparisons were conducted using the Steel–Dwass test. Multiple regression analysis with the least-squares method was performed to factorize factors related to FT and physician ISL, transforming them into continuous quantities. In all analyses, a p-value of < 0.05 was considered statistically significant.

## Results

### Questionnaire collection results

We received 1558 responses from patients and 825 responses from physicians (Fig. 1A).

### Patient background factors classified and summarized by PCA

Patient background factors were classified into six groups based on the questionnaire items, and PCA was performed on each group to identify PCs and subgroups. This resulted in 20 background factors, including two items not subject to PCA (Table S1). Group A consisted of seven patient attribute-related items, yielding three subgroups (age, visit, and location) and one basic item (location [population]) (Fig. S1A). The PCs were “age,” “location (longitude),” and “burden of access” (Fig. S1B–D). Group B had four income-related items, with PC’s “income” and “family support” (Fig. S1E). Group C, comprising six payment and insurance-related items, produced PCs “recent payment,” “out-of-pocket payment (ratio),” and “out-of-pocket payment (total)” (Fig. S1G and H). Group D contained 17 treatment-related items and led to four subgroups (stage, breast surgery, radiation therapy, and oncoplastic surgery) and other items (Fig. S2A). The PCs were “stage,” “breast surgery,” “oncoplastic surgery,” “radiation therapy,” and “related departments” (Fig. S2B–F). Group E included seven current diagnosis and treatment status-related items, resulting in PCs “endocrine therapy,” “chemotherapy,” and “frequency of visits” (Fig. S2G and H). Last, Group F’s two clinic time and medical cost-related items led to PCs, “consultation time (total)” and “consultation time (about cost)” (Fig. S1F).

### The distribution of COST and the grading of FT

**Fig. 2:**
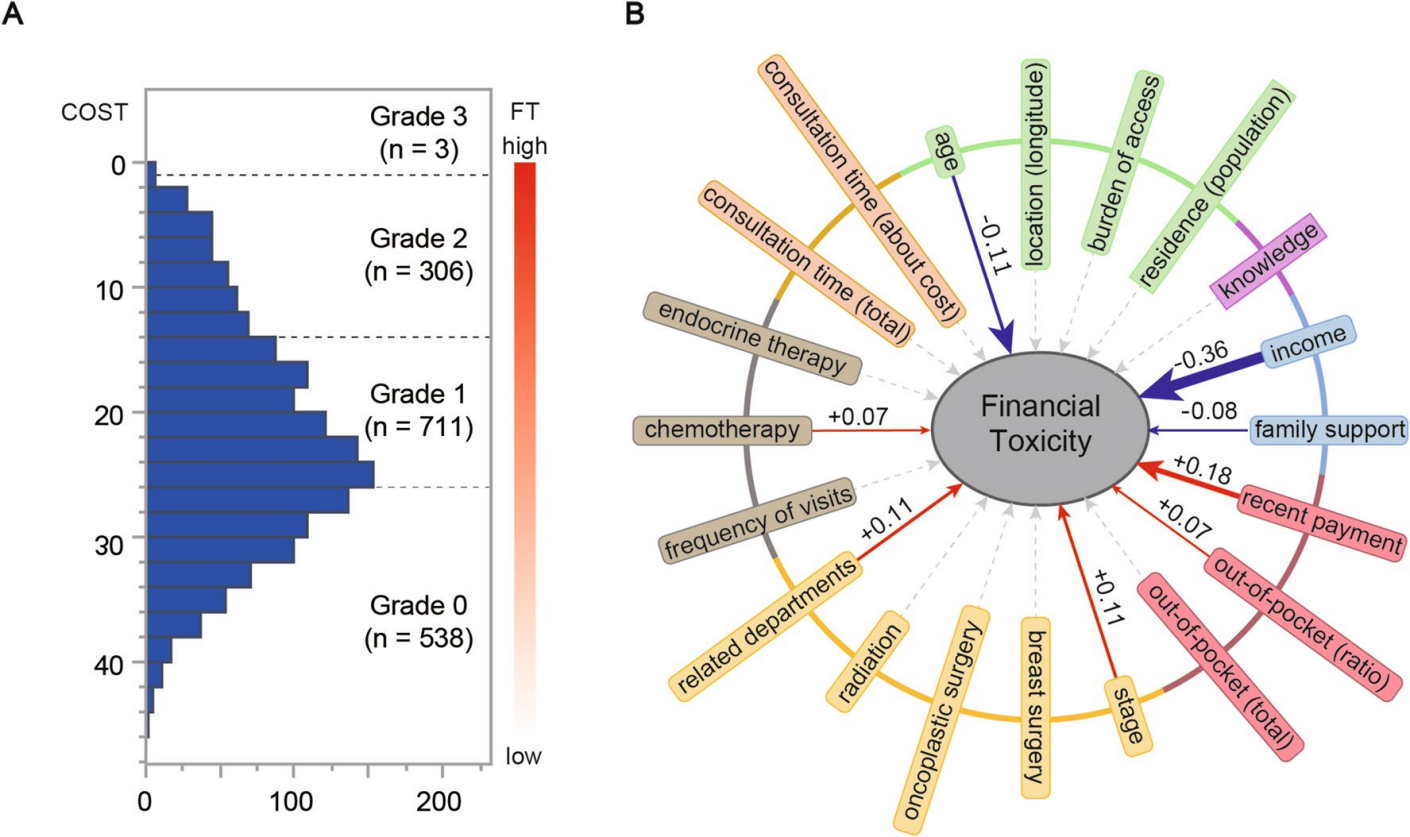
Patient financial toxicity (FT) and background factors. **A** Histogram of FTs and the number of cases per grade of toxicity. **B** Results of multiple regression analysis of FTs. Standard partial regression coefficients for significant items are presented. Items that are principal components are rounded at the corners. In the diagram, red arrows indicate a positive effect, while blue arrows indicate a negative effect. The thickness of the arrows is proportional to the degree of the effect

The mean value of the COST was 21.45, with a standard deviation of 8.81 (n = 1557; missing n = 1). The standard error of the mean was 0.223 (95% CI: 21.01–21.89). Based on the COST values, the patients were classified into four FT grades—Grade 0 (n = 583), Grade 1 (n = 711), Grade 2 (n = 306), and Grade 3 (n = 3) (Fig. 2A).

### Factors affecting FT

In order of importance, “recent payment” (β = 0.18, p < 0.0001), “stage” (β = 0.11, p < 0.0001), “related departments” (β = 0.11, p < 0.0001), “out-of-pocket payment (ratio)” (β = 0.07, p = 0.004), and “chemotherapy” (β = 0.07, p = 0.016) were found to have a positive effect on FT, while “income” (β =  − 0.36, p < 0.0001), “age” (β =  − 0.11, p < 0.0001), and “family support” (β =  − 0.08, p = 0.0001) were found to have a negative effect on FT, as revealed by the multiple regression analysis (Fig. 2B, Table 1).

**Table 1:** Multiple regression analysis for financial toxicity

Explanatory variable	Estimated value*	Lower 95%	Upper 95%	β**	p-value (Prob >|t|)	VIF†
Intercept	23.13	21.96	24.30	0.00	< 0.0001	
Age	– 0.75	– 1.06	– 0.44	– 0.11	< 0.0001	1.07
Location (longitude)	– 0.07	– 0.39	0.26	– 0.01	0.681	1.06
Burden of access	0.19	– 0.16	0.53	0.03	0.290	1.05
Residence (population)	0.00	0.00	0.00	– 0.03	0.190	1.12
Income	– 2.01	– 2.29	– 1.74	– 0.36	< 0.0001	1.15
Family support	– 0.61	– 0.97	– 0.25	– 0.08	0.001	1.02
Recent payment	1.46	1.06	1.85	0.18	< 0.0001	1.14
Out-of-pocket (ratio)	0.60	0.19	1.01	0.07	0.004	1.09
Out-of-pocket (total)	0.14	– 0.36	0.64	0.01	0.583	1.02
Stage	0.83	0.42	1.25	0.11	< 0.0001	1.41
Breast surgery	0.09	– 0.35	0.54	0.01	0.689	1.20
Oncoplastic surgery	– 0.25	– 0.59	0.09	– 0.04	0.153	1.19
Radiation	– 0.06	– 0.41	0.29	– 0.01	0.741	1.24
Related departments	0.82	0.45	1.20	0.11	< 0.0001	1.20
Endocrine therapy	0.09	– 0.25	0.43	0.01	0.607	1.07
Chemotherapy	0.51	0.10	0.92	0.07	0.016	1.35
Frequency of visits	0.10	– 0.31	0.52	0.01	0.632	1.07
Consultation time (total)	0.04	– 0.32	0.41	0.01	0.824	1.05
Consultation time (about cost)	– 0.05	– 0.54	0.44	– 0.01	0.830	1.02
Knowledge	– 0.20	– 0.60	0.19	– 0.02	0.310	1.05

*Partial regression coefficients were noted

**Standardized partial regression coefficients are noted

†VIF variance inflation factor

### Patients’ and physicians’ attitudes

**Fig. 3:**
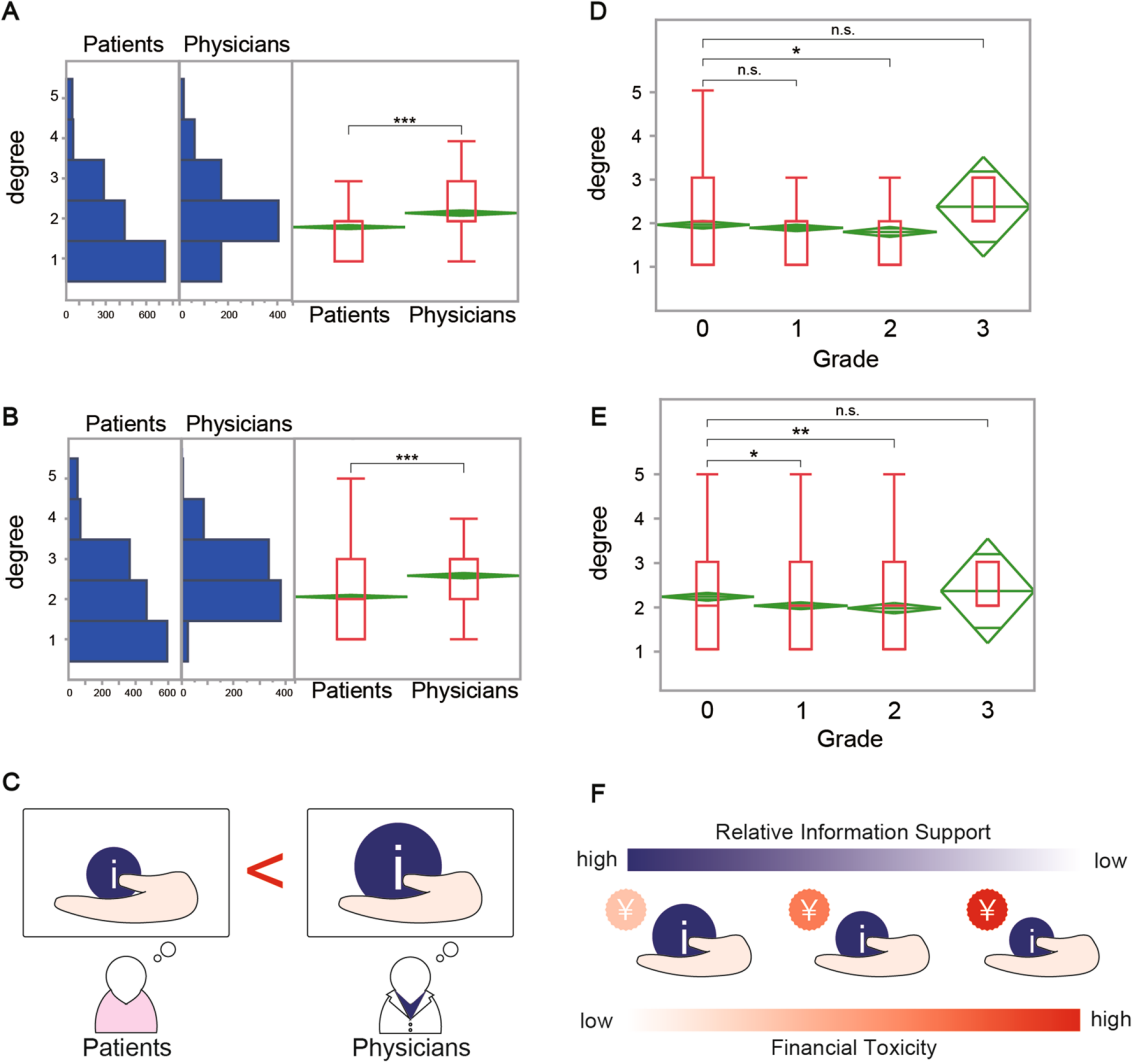
Frequency of explanations and opportunities to ask questions about medical expenses. **A** Difference between patients’ and physicians’ perceptions of the frequency of explanations. **B** Differences between patients’ and physicians’ perceptions of opportunities to ask questions. **C** Current perception of medical cost information support among patients and physicians. **D** Patients’ perceptions of the frequency of explanations by grade of Financial toxicity (FT). **E** Patients’ perceptions of opportunities to ask questions by grade of FT. **F** A diagram of the situation in which increased FT does not increase information support

In the evaluation of information support for medical costs, a clear inversion was found in response patterns between patients and physicians on the five-point Likert scale. The most frequent response for patients was “not at all,” whereas, for physicians, it was “somewhat agree,” indicating a discrepancy in perceived information support. Significant differences were observed between the two groups (p < 0.0001) (Fig. 3A). Similar response patterns and significant differences (p < 0.0001) were found regarding the opportunities to raise questions about medical costs and financial burdens (Fig. 3B).

### Survey findings on financial toxicity and information support

Differences were observed in the frequency of explanations and opportunities to ask questions about medical costs across FT grades. However, higher FT grades did not correspond to better information support for medical costs. For all grades, except for Grade 3, the most frequent responses skewed toward “not at all” and “somewhat agree.” There was a slight decrease in information support as the FT grade increased (Fig. 3D and E). The analysis revealed significant differences in the frequency of explanations (p = 0.0366) (Fig. 3D) and the opportunity to ask questions (p = 0.0009) (Fig. 3E) across the grades. Pairwise comparisons showed significant differences between Grades 2 and 0 (p = 0.0448) for the frequency of explanations (Fig. 3D), and between Grades 1 and 0 (p = 0.0017) and Grades 2 and 0 (p = 0.0109) for the opportunity to ask questions (Fig. 3E).

### Regional bias in ISL

**Fig. 4:**
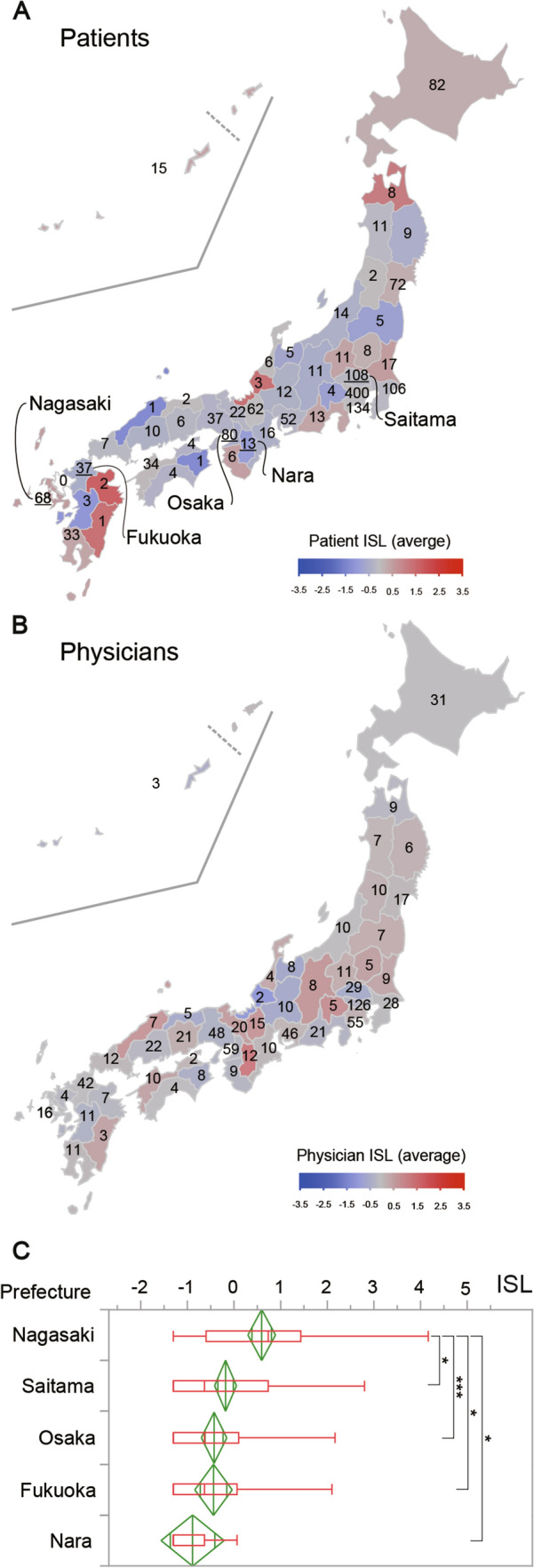
Comparison of patient and physician information support level (ISL) by Prefecture and multiple comparisons of patient ISLs. **A** Heatmap depicting the average patient ISLs by prefecture. **B** Heatmap illustrating the average physician ISLs by prefecture. For both (**A**) and (**B**), red and blue represent higher and lower ISLs, respectively, and the number of respondents per prefecture is shown on the map. **C** Multiple comparisons of patient ISLs are displayed, focusing exclusively on prefectures with significant differences. The vertical line within the box indicates the median of the sample; the two box ends represent the 25th and 75th percentile quartiles; and the whisker ends correspond to the maximum and minimum values, excluding outliers. The diamond symbolizes the mean and the 95% confidence interval of the mean

The ISLs for each patient and physician were calculated (Fig. S3A and B, Table S1, and Table S3), and their distribution across Japan was found to be non-normal (Fig. S3C and D). Despite no apparent bias toward specific regions as indicated by the mapping of patient ISLs, a difference in the distribution of ISLs by prefecture was observed (Fig. 4A). In contrast, such regional trends were not clear in the mapping of physician ISLs (Fig. 4B). Multiple comparisons of ISLs between prefectures detected a significant difference for patients (p < 0.0001) but not for physicians (p = 0.234). Further pairwise comparisons for patients identified significant differences in ISL exclusively between Nagasaki and Osaka (p < 0.001), Saitama (p = 0.019), Fukuoka (p = 0.023), and Nara (p = 0.039) prefectures (Fig. 4C).

### Classification and summary of physician background factors using PCA and correlation analysis

PCA was performed on latitude and longitude to summarize them as “location (longitude)” (Fig. S1D, Table S3). For the other basic items, we observed variance inflation factor values of three or higher for age and years after graduation when performing multiple regression analyses without confounding (Table S2). To avoid multicollinearity, a correlation analysis was conducted between factors (Fig. S4A) and found correlations between age, years after graduation, and gender (male) (r = 0.85 and 0.44, respectively). PCA was performed on these factors, resulting in PCs being designated as “age”, “gender (male)”, and “experience”, and confounding was removed (Fig. S4B and C, Table S3). The correlation coefficient between the other factors was |r|< 0.25. In summary, 12 background factors were identified, including 4 PCs and 8 basic items for which no PCA was performed (Table S3).

### Factors affecting physician ISL

**Fig. 5:**
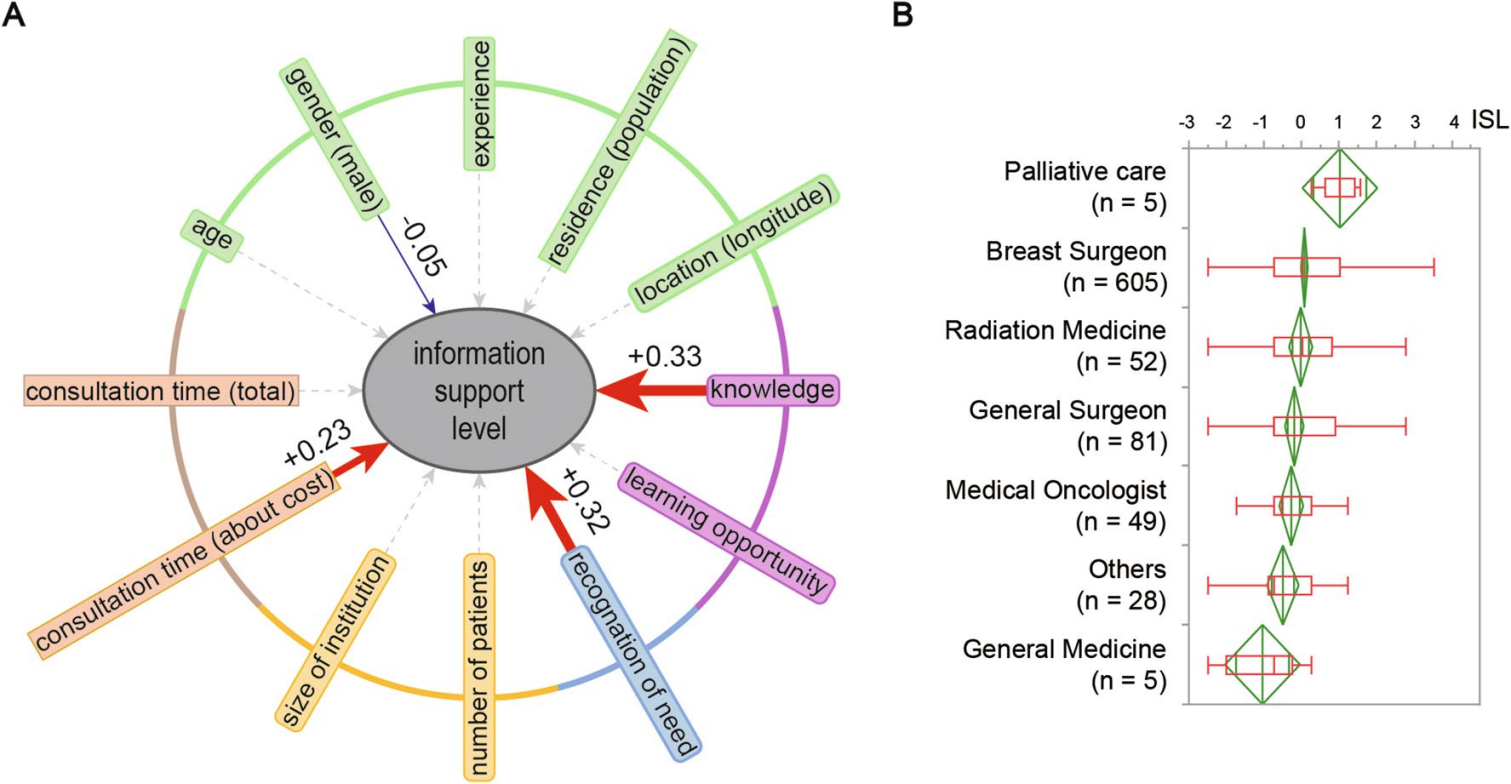
Physician information support level (ISL) by Specialty and background factors. **A** Results of multiple regression analysis of ISL. Standard partial regression coefficients for significant items are presented. Items that are principal components are rounded corners. In the diagram, red arrows indicate a positive effect, while blue arrows indicate a negative effect. The thickness of the arrows is proportional to the degree of the effect. **B** Differences in physician ISLs among specialties are presented. The sample size is denoted in parentheses. The vertical line in the box indicates the median of the sample; the two ends are the 25th and 75th percentile quartiles; and the maximum and minimum excluding outliers are the two ends of the whiskers. The diamonds represent the mean and the 95% confidence interval of the mean

The multiple regression analysis revealed that, in order of importance, knowledge (β = 0.33, p < 0.0001), recognition of need (β = 0.32, p < 0.0001), and consultation time (about cost) (β = 0.23, p < 0.0001) had a positive effect on ISL. However, “gender (male)” (β =  − 0.06, p = 0.029) had a negative effect on ISL (Fig. 5A, Table 2).

**Table 2:** Multiple regression analysis for information support level of physician

Explanatory variable	Estimated value*	Lower 95%	Upper 95%	β**	p-value (Prob >|t|)	VIF†
Intercept	– 3.26	– 3.65	– 2.86	0.00	< 0.0001	
Age	– 0.04	– 0.09	0.00	– 0.05	0.071	1.14
Gender (male)	– 0.09	– 0.16	– 0.01	– 0.06	0.029	1.03
Experience	0.01	– 0.15	0.17	0.00	0.890	1.02
Location (longitude)	0.01	– 0.04	0.06	0.01	0.679	1.04
Residence (population)	0.00	0.00	0.00	– 0.05	0.084	1.07
Size of institution	0.00	– 0.04	0.04	0.00	0.999	1.14
Number of patients	0.01	– 0.04	0.05	0.01	0.688	1.07
Consultation time (total)	– 0.02	– 0.07	0.04	– 0.01	0.613	1.11
Consultation time (about cost)	0.45	0.34	0.56	0.23	< 0.0001	1.07
Knowledge	0.44	0.36	0.52	0.33	< 0.0001	1.20
Learning opportunity	0.10	– 0.03	0.23	0.04	0.130	1.05
Recognation of need	0.36	0.30	0.43	0.32	< 0.0001	1.09

*Partial regression coefficients were noted

**Standardized partial regression coefficients are noted

†VIF Variance inflation factor

### Comparison of ISL by physician specialty

The Kruskal–Wallis’s test for differences in the distribution of ISL by specialty revealed significant differences (p < 0.0022), but the Steel–Dwass test revealed no statistically significant differences for any of the combinations (Fig. 5B).

## Discussion

This study examined the financial burden faced by patients with breast cancer in Japan. The findings revealed that the most recent payment significantly impacts patients’ financial burden. Furthermore, the study found that FT tended to be higher among patients with more advanced cancer stages, requiring multispecialty interventions such as anticancer therapy. This trend was also observed in younger patients and those with lower incomes. Notably, the study identified a discrepancy in the perceived level of informational support between patients and physicians. Patients often felt they received less support than they thought their perceptions of the support provided by the physicians (Fig. 3C). Furthermore, the results suggested that as the financial burden grows, patients’ perception of information support tends to diminish (Fig. 3F). From the viewpoint of physicians’ perception of information support, factors such as their knowledge, perceived necessity, and time spent discussing costs were significant contributors. This finding suggests that physicians who acknowledge the importance of informational support and have a comprehensive understanding of medical costs may be better equipped to provide more substantial cost-related support. Regional disparities in patients’ perceived level of informational support were found, with patients in some prefectures feeling they received more support than others did. However, it is crucial to note that this study focused on a select number of facilities, suggesting that facility-specific differences might exert a greater influence than geographic factors. In terms of physician ISL across specialties, a significant overall difference was found. However, no specific pairs of specialties were identified as having statistically significant differences in subsequent pairwise comparisons. These results should be interpreted with caution due to potential biases related to sample sizes and multiple comparisons involving smaller sample size groups [29].

This study effectively used PCA, a useful technique for reducing large data sets to their core components, thereby avoiding multicollinearity [30]. By summarizing multiple survey items, the study offers a clear insight into the economic burden faced by breast cancer patients. The study’s limitations include the use of survey items about patients’ and physicians’ attitudes, which cannot be used as an objective measure. In addition, we made no comparisons between the pairs of physicians and patients surveyed, and the examination of underlying factors was limited to an examination of associations within the same sample.

Japan has a universal health insurance system where citizens pay insurance premiums to cover most medical expenses, with patients typically paying up to 30% of the costs. Moreover, the high-cost medical care benefit system provides a maximum monthly amount based on the insured person’s age and income level, which varies with the highest being approximately 252,600 JPY for a person under 70 years old with an average annual income of approximately 11.6 million JPY or more [31]. Despite this, there is a three-month lag between the time of application and receipt of benefits, which can put a significant financial strain on households, especially on younger generations with limited savings. Furthermore, some physicians may not be familiar with the high-cost medical care benefits system, and patients may lack information support during the application process.

In future directions, refining hospital systems to enhance support for patients regarding medical costs and fostering cooperation among medical personnel is crucial. This can alleviate patients’ financial burden by leveraging existing resources and expertise and strengthening collaboration among physicians, medical social workers, nurses, and other healthcare professionals [32]. It is also essential to engage with nonprofit organizations and patient associations for opinion exchange and social awareness initiatives. Furthermore, offering seamless information support for post-treatment financial burdens tailored to individual cases is crucial [33]. For example, providing patients with information about the maximum-cost-applicable certificate system through physicians or hospital staff may prevent a three-month reimbursement delay. Some studies have evaluated the effectiveness of tools like “ChemoCalc™,” which calculates drug costs in offering financial information support to patients [34]. Other research has investigated patients’ willingness to pay [35] and the economic impact of biosimilars on out-of-pocket expenses [36]. Physicians need to be aware of the extent to which proposed treatments impose financial burdens on patients and to consider better-personalized treatment options.

## Conclusion

This study highlights the issue of FT in patients with breast cancer in Japan and emphasizes the significance of addressing the financial burden associated with recent payments. It also identifies the need for improved information support for medical costs and a deeper understanding by physicians of the financial impact of proposed treatments. Addressing these challenges at both institutional and individual levels is crucial. Collaborative efforts among diverse professions are vital for alleviating FT in patients with breast cancer, optimizing existing resources and expertise, and delivering personalized information support.

## Supplementary Information

Below is the link to the electronic supplementary material.

Supplementary file1 (XLSX 18 KB)

Supplementary file2 Fig. S1 Principal Component Analysis diagram summarizing patient background factors (Fig. S1D is common with physicians) Fig. S2 Principal Component Analysis diagram summarizing patient background factors Fig. S3 Information Support Level (ISL) for Patients and Physicians. (A) Definition of ISL for patients. (B) Definition of ISL for physicians. (C) Histogram of ISL for patients. (D) Histogram of ISL for physicians Fig. S4 Correlations among Physician Questions and Confounding Factors. (A) Correlation coefficients are presented in the heatmap, with red and blue indicating a positive and negative correlation, respectively. (B) Principal component 1 (PC1) and Principal component 2 (PC2) between age, years after graduation, and gender (male). (C) PC1 and Principal component 3 (PC3) between age, years after graduation, and gender (male) (PDF 683 KB)

## References

[1] DeSantis CE, Bray F, Ferlay J, Lortet-Tieulent J, Anderson BO, Jemal A (2015). International variation in female breast cancer incidence and mortality rates. Cancer Epidemiol Biomarker Prev.

[2] Harbeck N, Gnant M (2017). Breast cancer. Lancet.

[3] Ohuchi N, Suzuki A, Sobue T, Kawai M, Yamamoto S, Zheng YF (2016). Sensitivity and specificity of mammography and adjunctive ultrasonography to screen for breast cancer in the Japan strategic anti-cancer randomized trial (J-START): a randomised controlled trial. Lancet.

[4] Trieu PDY, Mello-Thoms CR, Barron ML, Lewis SJ (2022). Look how far we have come: BREAST cancer detection education on the international stage. Front Oncol.

[5] McDonald ES, Clark AS, Tchou J, Zhang P, Freedman GM (2016). Clinical diagnosis and management of breast cancer. J Nucl Med.

[6] Gabriel CA, Domchek SM (2010). Breast cancer in young women. Breast Cancer Res.

[7] Miller KD, Fidler-Benaoudia M, Keegan TH, Hipp HS, Jemal A, Siegel RL (2020). Cancer statistics for adolescents and young adults, 2020. CA Cancer J Clin.

[8] Kim J, Rajan SS, Du XL, Franzini L, Giordano SH, Morgan RO (2018). Association between financial burden and adjuvant hormonal therapy adherence and persistent use for privately insured women aged 18–64 years in BCBS of Texas. Breast Cancer Res Treat.

[9] Offodile AC, Asaad M, Boukovalas S, Bailey C, Lin YL, Teshome M (2021). Financial toxicity following surgical treatment for breast cancer: a cross-sectional pilot study. Ann Surg Oncol.

[10] Dean LT, Moss SL, Rollinson SI, Frasso Jaramillo L, Paxton RJ, Owczarzak JT (2019). Patient recommendations for reducing long-lasting economic burden after breast cancer. Cancer.

[11] Thom B, Benedict C, Friedman DN, Kelvin JF (2018). The intersection of financial toxicity and family building in young adult cancer survivors. Cancer.

[12] Meernik C, Mersereau JE, Baggett CD, Engel SM, Moy LM, Cannizzaro NT (2022). Fertility preservation and financial hardship among adolescent and young adult women with cancer. Cancer Epidemiol Biomarkers Prev.

[13] Kamal KM, Covvey JR, Dashputre A, Ghosh S, Shah S, Bhosle M, Zacker C (2017). A systematic review of the effect of cancer treatment on work productivity of patients and caregivers. J Manag Care Spec Pharm.

[14] Allaire BT, Ekwueme DU, Guy GP, Li C, Tangka FK, Trivers KF (2016). Medical care costs of breast cancer in privately insured women aged 18–44 years. Am J Prev Med.

[15] Han S, Jang BH, Suh HS, Hwang DS (2019). Complementary medicine use and costs in patients with breast cancer who experienced treatment-related side effects: a cross-sectional survey in Korea. Complement Ther Med.

[16] Coughlin SS, Ayyala DN, Tingen MS, Cortes JE (2020). Financial distress among breast cancer survivors. Curr Cancer Rep.

[17] Jagsi R, Ward KC, Abrahamse PH, Wallner LP, Kurian AW, Hamilton AS (2018). Unmet need for clinician engagement regarding financial toxicity after diagnosis of breast cancer. Cancer.

[18] Goldstein DA (2017). Financial toxicity in cancer care-edging toward solutions. Cancer.

[19] Ehsan AN, Wu CA, Minasian A, Singh T, Bass M, Pace L (2023). Financial toxicity among patients with breast cancer worldwide: a systematic review and meta-analysis. JAMA Netw Open.

[20] Fischer KA, Walling A, Wenger N, Glaspy J (2020). Cost health literacy as a physician skill-set: the relationship between oncologist reported knowledge and engagement with patients on financial toxicity. Support Care Cancer.

[21] Kajimoto Y, Shibutani T, Nagao S, Yamaguchi S, Suzuki S, Mori M (2022). Validity of the comprehensive score for financial toxicity (COST) in patients with gynecologic cancer. Int J Gynecol Cancer.

[22] Japan GIAo. Longitude and latitude of prefectural government buildings and east, west, south, north and south end points. (https://www.gsi.go.jp/common/000230936.pdf)

[23] MIC Ministry of Internal Affairs and Communications J. The statistical handbook of Japan 2022. (https://www.stat.go.jp/english/data/handbook/index.html)

[24] de Souza JA, Yap BJ, Hlubocky FJ, Wroblewski K, Ratain MJ, Cella D, Daugherty CK (2014). The development of a financial toxicity patient-reported outcome in cancer: the cost measure. Cancer.

[25] de Souza JA, Yap BJ, Wroblewski K, Blinder V, Araújo FS, Hlubocky FJ (2017). Measuring financial toxicity as a clinically relevant patient-reported outcome: the validation of the comprehensive score for financial toxicity (COST). Cancer.

[26] Honda K, Gyawali B, Ando M, Sugiyama K, Mitani S, Masuishi T (2018). A prospective survey of comprehensive score for financial toxicity in Japanese cancer patients: report on a pilot study. Ecancermedicalscience.

[27] Honda K, Gyawali B, Ando M, Kumanishi R, Kato K, Sugiyama K (2019). Prospective survey of financial toxicity measured by the comprehensive score for financial toxicity in Japanese patients with cancer. J Glob Oncol.

[28] Kajimoto Y, Honda K, Suzuki S, Mori M, Tsubouchi H, Nakao K (2023). Association between financial toxicity and health-related quality of life of patients with gynecologic cancer. Int J Clin Oncol.

[29] Resnicow K, Patel MR, McLeod MC, Katz SJ, Jagsi R (2019). Physician attitudes about cost consciousness for breast cancer treatment: differences by cancer sub-specialty. Breast Cancer Res Treat.

[30] Takahashi T, Kawasaki S. Survey and virtual experimentation through questionnaires (2nd ed). JUSE Press; 2021.

[31] Ministry of Health Law, Japan. To all users of the high-cost medical care benefit system. (https://www.mhlw.go.jp/content/000333279.pdf)

[32] Smith GL, Banegas MP, Acquati C, Chang S, Chino F, Conti RM (2022). Navigating financial toxicity in patients with cancer: a multidisciplinary management approach. CA Cancer J Clin.

[33] Khera N, Holland JC, Griffin JM (2017). Setting the stage for universal financial distress screening in routine cancer care. Cancer.

[34] Kuba S, Moriuchi H, Yamanouchi K, Shibata K, Yano H, Oikawa M (2021). Protocol for studying the efficiency of ChemoCalc software in helping patients to understand drug treatment costs for breast cancer: a multicenter, open-label, randomized phase 2 study. Contemp Clin Trials Commun.

[35] Iwatani T, Hara F, Shien T, Sasaki K, Katayama H, Fukuda H (2021). Prospective observational study estimating willingness-to-pay for breast cancer treatments through contingent valuation method in Japanese breast cancer patients (JCOG1709A). Jpn J Clin Oncol.

[36] Hara F, Tajima K, Tanabe K (2019). Current situation and challenges regarding biosimilars in Japan: an example of trastuzumab biosimilars for breast cancer. Future Oncol.

