# Correction: Factors associated with financial toxicity in patients with breast cancer in Japan: a comparison of patient and physician perspectives

**DOI:** 10.1007/s12282-023-01479-w

**Published:** 2023-07-03

**Authors:** Sumito Saeki, Tsuguo Iwatani, Atsuko Kitano, Naomi Sakurai, Yuko Tanabe, Chikako Yamauchi, Ataru Igarashi, Yusuke Kajimoto, Sayaka Kuba, Fumikata Hara, Yasuaki Sagara, Shinji Ohno, Collaborative Study Group of Scientific Research of the Japanese Breast Cancer Society

**Affiliations:** 1 Breast Oncology Center, Cancer Institute Hospital, Japanese Foundation for Cancer Research, 3-8-31 Ariake, Koto-ku, Tokyo, 135-8550 Japan; 2 Graduate School of Medicine, Department of Cancer Therapy and Surgery, Tohoku University, Sendai, Japan; 3 Department of Breast Surgery, National Cancer Center Hospital East, Chiba, Japan; 4 Department of Medical Oncology, St Luke’s International Hospital, Tokyo, Japan; 5 Cancer Solutions, Inc, Tokyo, Japan; 6 Department of Medical Oncology, Toranomon Hospital, Tokyo, Japan; 7 Radiation Therapy Center, Shiga General Hospital, Moriyama, Japan; 8 Department of Pharmaceutical Policy, The University of Tokyo, Tokyo, Japan; 9 Graduate School of Pharmaceutical Sciences, University of Tokyo, Tokyo, Japan; 10 Oncology Science Unit, MSD K.K, Tokyo, Japan; 11 Graduate School of Biomedical Sciences, Department of Surgery, Nagasaki University, Nagasaki, Japan; 12 Department of Breast and Thyroid Surgical Oncology, Hakuaikai Sagara Hospital, Kagoshima, Japan

**Correction: Breast Cancer** 10.1007/s12282-023-01476-z

In the original publication of the article, the last author’s name “Shinji Ohno” was deleted and the first author name “Sumito Saeki” was repeated instead during correction stage. The original article has been updated.

The publisher sincerely apologizes for any inconvenience this error may have caused.

