# Supplementary figures and images for: Factors associated with financial toxicity in patients with breast cancer in Japan: a comparison of patient and physician perspectives
Source: Breast Cancer. 2023 Jun 13;30(5):820–30. doi: 10.1007/s12282-023-01476-z (PMC10404186; doi:10.1007/s12282-023-01476-z)

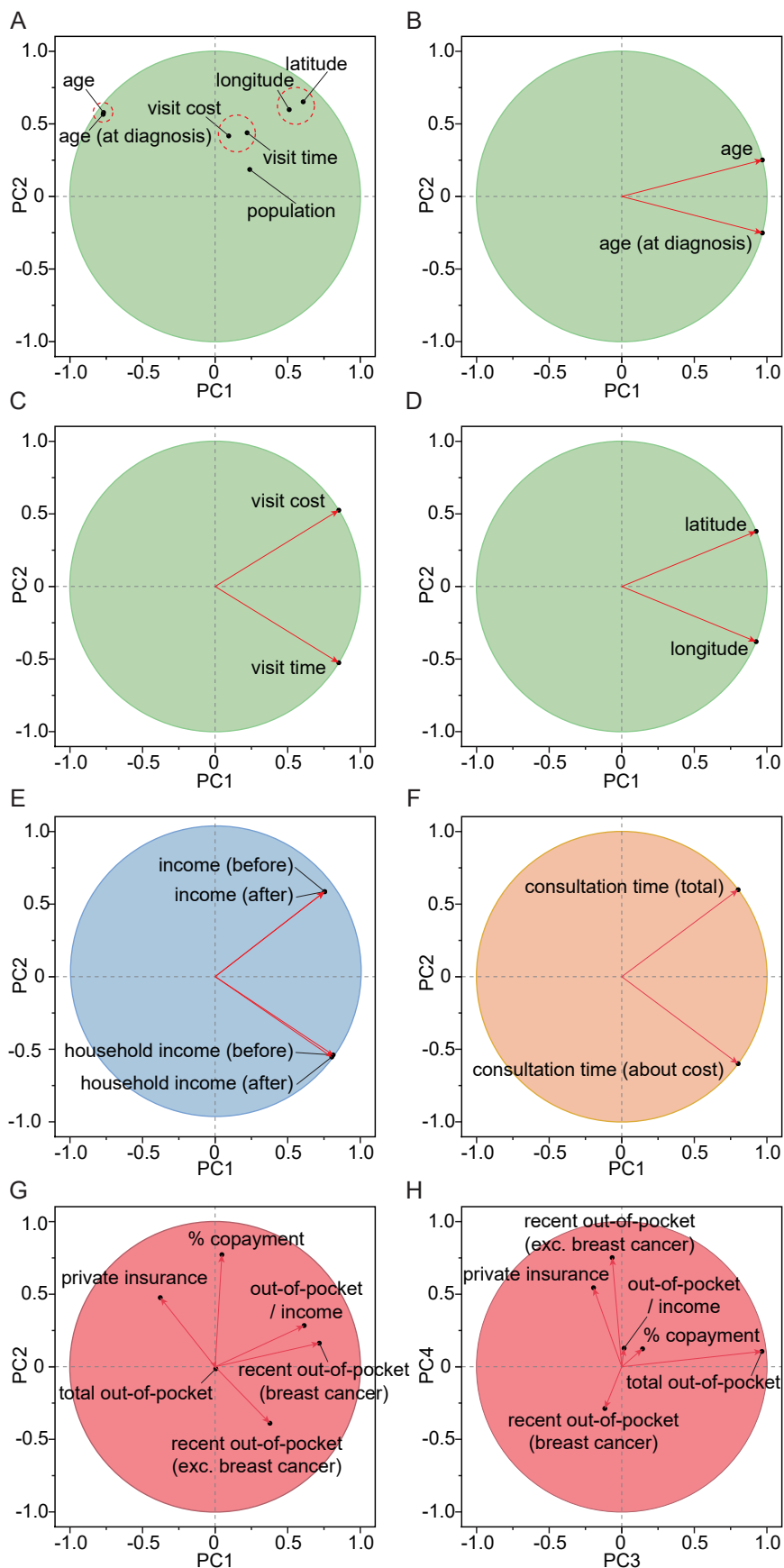

Supplement Fig.1

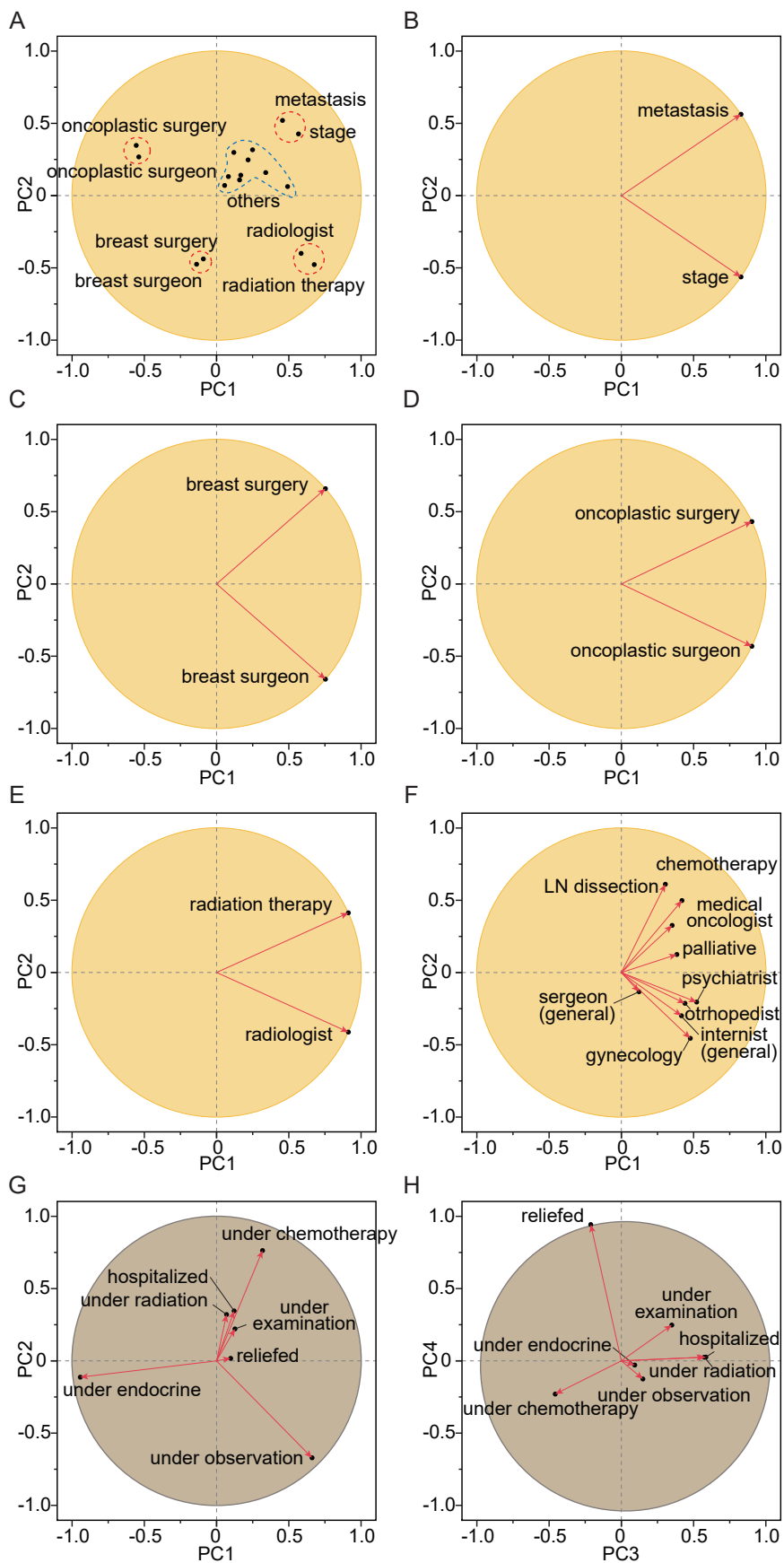

Supplement Fig.2

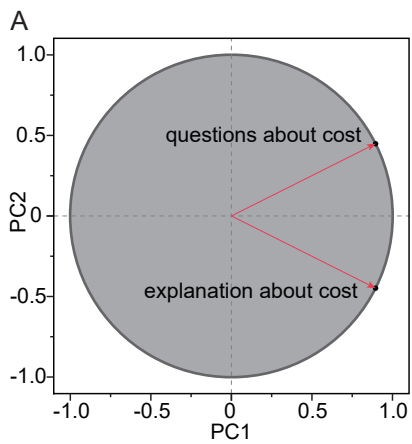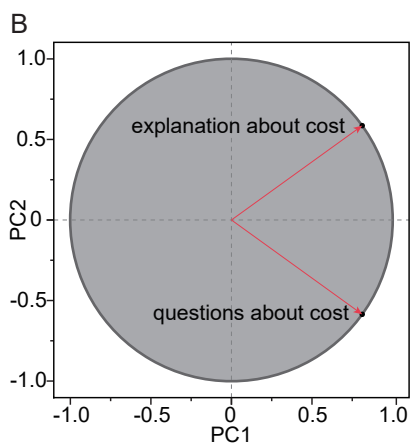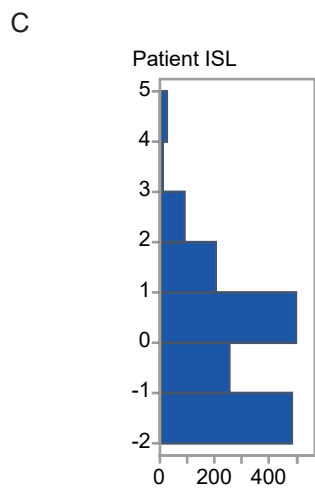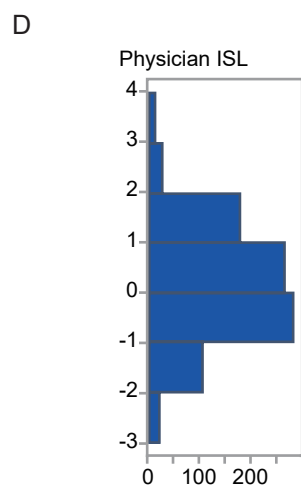

A

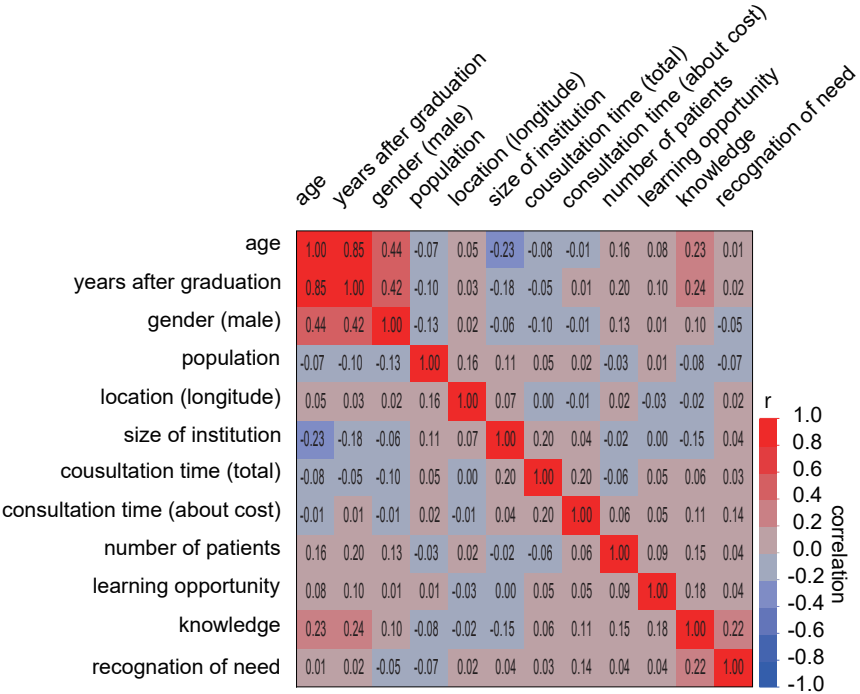

B

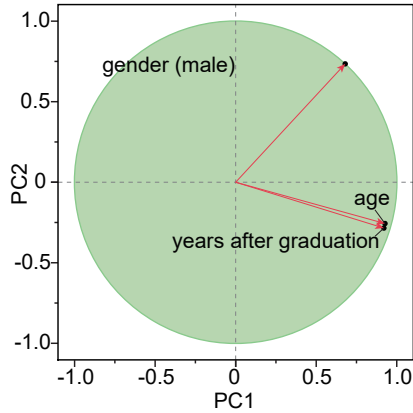

C

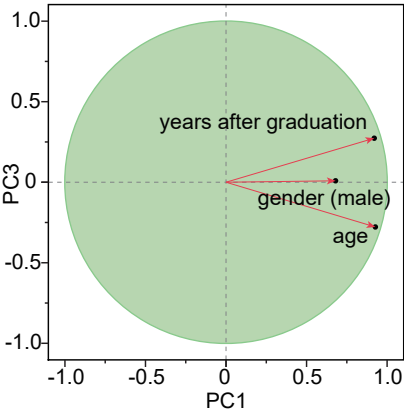

Supplement: Supplementary file 2 — Supplementary file2 Fig. S1 Principal Component Analysis diagram summarizing patient background factors (Fig. S1D is common with physicians) Fig. S2 Principal Component Analysis diagram summarizing patient background factors Fig. S3 Information Support Level (ISL) for Patients and Physicians. (A) Definition of ISL for patients. (B) Definition of ISL for physicians. (C) Histogram of ISL for patients. (D) Histogram of ISL for physicians Fig. S4 Correlations among Physician Questions and Confounding Factors. (A) Correlation coefficients are presented in the heatmap, with red and blue indicating a positive and negative correlation, respectively. (B) Principal component 1 (PC1) and Principal component 2 (PC2) between age, years after graduation, and gender (male). (C) PC1 and Principal component 3 (PC3) between age, years after graduation, and gender (male) (PDF 683 KB) [file 12282_2023_1476_MOESM2_ESM.pdf]
